# Tiansi Liquid Modulates Gut Microbiota Composition and Tryptophan–Kynurenine Metabolism in Rats with Hydrocortisone-Induced Depression

**DOI:** 10.3390/molecules23112832

**Published:** 2018-10-31

**Authors:** Dan Cheng, Hongsheng Chang, Suya Ma, Jian Guo, Gaimei She, Feilong Zhang, Lingling Li, Xinjie Li, Yi Lu

**Affiliations:** 1 School of Chinese Medicine, Beijing University of Chinese Medicine, Beijing 100029, China; chengdan@bucm.edu.cn (D.C.); masuya0217@163.com (S.M.); guojian323@sina.com (J.G.); delko@bucm.edu.cn (F.Z.); 20170931145@bucm.edu.cn (L.L.); lixinjie@bucm.edu.cn (X.L.); 2 School of Chinese Materia Medica, Beijing University of Chinese Medicine, Beijing 102488, China; chs1971@sina.com (H.C.); shegaimei@126.com (G.S.)

**Keywords:** Tiansi Liquid, antidepressant effects, 16S rRNA high-throughput pyrosequencing, gut microbiota, high-performance liquid chromatography-mass spectrometry, tryptophan–kynurenine pathway

## Abstract

Tiansi Liquid is a traditional Chinese herbal medicine used to treat depression; however, the underlying mechanisms remain unclear. Here, we examined the effect of Tiansi Liquid in a rat model of hydrocortisone-induced depression using behavioral testing, 16S rRNA high-throughput pyrosequencing and high-performance liquid chromatography-mass spectrometry-based metabolomics of the tryptophan (TRP)–kynurenine (KYN) pathway. Tiansi Liquid significantly improved the sucrose preference and exploratory behavior of the depressive rats. The richness of intestinal mucosa samples from the model (depressive) group tended to be higher than that from the control group, while the richness was higher in the Tiansi Liquid-treated group than in the model group. Tiansi Liquid increased the relative abundance of some microbiota (*Ruminococcaceae*, *Lactococcus*, *Lactobacillus*, *Lachnospiraceae_NK4A136_group*). Metabolomics showed that Tiansi Liquid reduced the levels of tryptophan 2,3 dioxygenase, indoleamine 2,3-dioxygenase, quinoline and the KYN/TRP ratio, while increasing kynurenic acid and 5-HT levels. Correlation analysis revealed a negative relationship between the relative abundance of the *Lachnospiraceae_NK4A136_group* and quinoline content. Collectively, these findings suggest that Tiansi Liquid ameliorates depressive symptoms in rats by modulating the gut microbiota composition and metabolites in the TRP–KYN pathway.

## 1. Introduction

Depression is a common psychiatric illness characterized by low mood, loss of interest in activities, and somatic symptoms [1,2]. Many factors are associated with depression, including biochemical, psychological, genetic and social factors [3,4,5]. Chronic stress is considered the major risk factor for depression [6], and there is accumulating evidence that gut microbiota plays an important role in the disorder [7,8,9]. Indeed, gut microbiota can regulate the stress response [10], while stress can change the composition of gut microbiota [11,12].

Gut microbiota signals the brain by regulating tryptophan metabolism and the kynurenic acid and quinolinic acid of the downstream metabolites [13,14,15]. In addition, the tryptophan–kynurenine (TRP–KYN) metabolic pathway plays an important role in the development of depression. Tryptophan is the precursor of serotonin (5-HT), and tryptophan deficiency can induce depression-like behaviors in rats [16]. Kynurenine is upregulated in depressed patients [17]. Some other metabolites in the TRP–KYN pathway, such as quinoline (QUIN), are neurotoxic, while kynurenic acid (KYNA) is neuroprotective [18]. Meanwhile, the neurotoxic metabolite synthesized by kynurenine monooxygenase, 3-hydroxykynurenine (3-HK), its upregulation in inflamed brain and the potential capability of medicinal plants and extracts to downregulate it restoring 5-HT signaling [19]. Previous studies show that changes in gut microbiota may result in the development of depression by affecting metabolites in the TRP–KYN pathway and reducing 5-HT levels [20].

Tiansi Liquid is a traditional Chinese medicine often prescribed for treating depression in China [21,22,23,24,25]. It was initially described in “Syndrome Differentiation Records”, which was compiled by Shi-yuan Chen during the Qing dynasty. Tiansi Liquid contains two herbs, *Morinda officinalis* and *Cuscuta chinensis*. There are many clinical studies demonstrating that *Morinda officinalis* capsules have therapeutic effects in depressed patients [21,22]. Furthermore, preclinical studies show that the methanolic extract of *Cuscuta chinensis* has an antidepressive effect in mice, significantly improving performance in the tail suspension test and the forced swimming test [24,25].

Our previous study showed that Tiansi Liquid inhibits the expression of indoleamine 2,3-dioxygenase (IDO), which is the rate-limiting enzyme in the TRP–KYN pathway [26], in the mice depression model caused by an intraperitoneal injection of lipopolysaccharides. Based on this previous study, we hypothesized that Tiansi Liquid might exert its antidepressive effect by modulating the TRP–KYN metabolic pathway. In the present study, we test this hypothesis by performing behavioral testing, 16S rRNA high-throughput pyrosequencing, and high-performance liquid chromatography-mass spectrometry (HPLC-MS)-based metabolomics of the TRP–KYN pathway in a rat model of hydrocortisone-induced depression.

## 2. Results

### 2.1. Model Validation and Behavioral Changes

**Figure 1:**
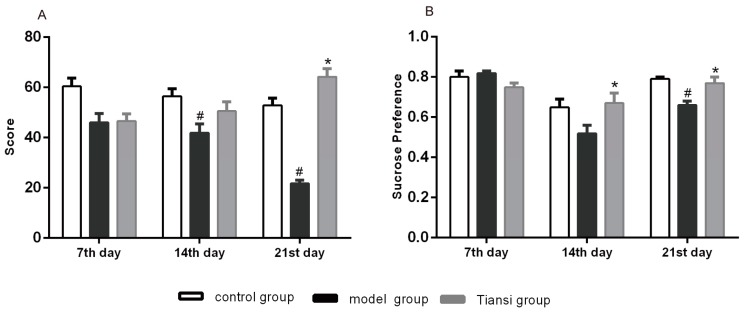
Effects of Tiansi Liquid treatment on the behavior of rats. Effects of Tiansi Liquid treatment on performance in the open field test (**A**) and sucrose preference test (**B**). Ten rats in each group. Statistical analyses were performed using one-way ANOVA and Tukey—Kramer post hoc comparisons. ^#^ *p* < 0.05, vs. control group; * *p* < 0.05, vs. model group.

The model group had a much lower score in the open field test at 14 days compared with the control group (*p* < 0.05) (Figure 1A), suggesting that we had successfully produced the rat model of hydrocortisone-induced depression. Meanwhile, the score in the Tiansi group was significantly increased compared with the model group in the third trial (*p* < 0.05) (Figure 1A). These results suggest that Tiansi Liquid increases exploratory behavior and activity in depressive rats.

Compared with the control group, the sucrose preference index in the model group was significantly lower on day 21 (*p* < 0.05) (Figure 1B). The sucrose preference index in the Tiansi-treated group was significantly increased compared with the model group on day 14 (*p* < 0.05) (Figure 1B), suggesting that Tiansi Liquid improve anhedonia condition of rats with depression.

### 2.2. Regulation of the TRP–KYN Metabolic Pathway

#### 2.2.1. Effects on TRP–KYN Pathway Metabolites

**Figure 2:**
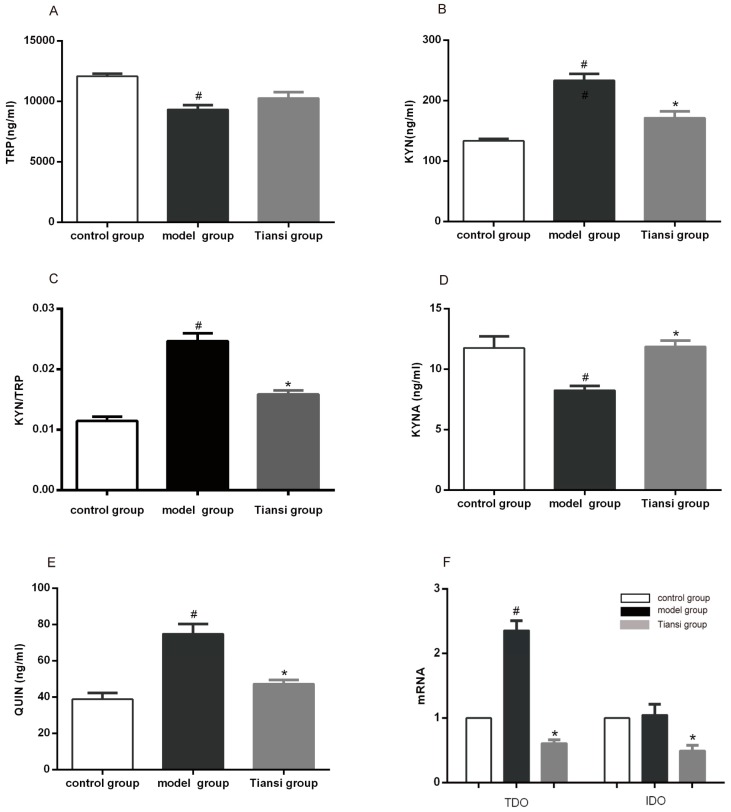
Effects of Tiansi Liquid on TRP–KYN metabolites. Effects of Tiansi Liquid treatment on TRP (**A**), KYN (**B**), KYN/TRP ratio (**C**), KYNA (**D**) and QUIN (**E**). Effect of Tiansi Liquid treatment on the expression of TDO mRNA in the liver and IDO mRNA in the hippocampus (**F**). TRP, KYN, KYNA and QUIN denote tryptophan, kynurenine, kynurenic acid and quinoline, respectively; TDO denotes tryptophan-2,3-dioxygenase; and IDO denotes indoleamine 2,3-dioxygenase. Ten rats in each group. Statistical analyses were performed using one-way ANOVA and Tukey—Kramer post hoc comparisons. ^#^ *p* < 0.05, vs. control group; * *p* < 0.05, vs. model group.

On day 21 after hydrocortisone injection, according to the results of the plasma samples were tested by HPLC-MS, the levels of KYN, QUIN and the KYN/TRP ratio were significantly increased in the model group compared with the control group (*p* < 0.05) (Figure 2B,C,E), while the levels of TRP and KYNA were significantly decreased (*p* < 0.05) (Figure 2A,D). In the Tiansi-treated group, the levels of KYN, QUIN and the KYN/TRP ratio were significantly decreased compared with the model group (*p* < 0.05) (Figure 2B,C,E), while the levels of KYNA were significantly higher than in the model group (*p* < 0.05) (Figure 2D). These results demonstrate that Tiansi Liquid regulates the TRP–KYN metabolic pathway.

#### 2.2.2. Effects on the Expression of Rate-Limiting Enzymes in the TRP–KYN Pathway

Tryptophan-2,3-dioxygenase (TDO) and indoleamine 2,3-dioxygenase (IDO) are important rate-limiting enzymes in the TRP–KYN metabolic pathway. TDO mRNA in the liver and IDO mRNA in the hippocampus were measured using real-time quantitative PCR. The levels of TDO mRNA were higher in the model group than in the control group (*p* < 0.05). Furthermore, Tiansi Liquid significantly decreased the expression of TDO mRNA in the liver and the expression of IDO mRNA in the hippocampus of depressive rats (*p* < 0.05) (Figure 2F). These results show that Tiansi Liquid inhibits the expression of the rate-limiting enzymes in the TRP-KYN pathway in rats with depression.

### 2.3. Effects on 5-HT_1A_ Receptor Expression and 5-HT Content

**Figure 3:**
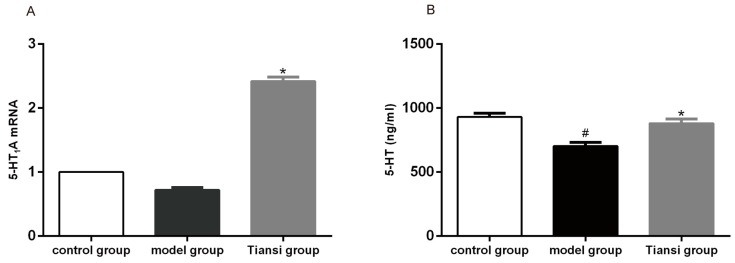
Effects of Tiansi Liquid on 5-HT_1A_ receptor expression and plasma 5-HT concentration. 5-HT_1A_ mRNA levels (**A**) and plasma 5-HT concentration (**B**) in the three groups. There were ten rats in each group. Statistical analyses were performed using one-way ANOVA and Tukey—Kramer post hoc comparisons. ^#^ *p* < 0.05, vs. control group; * *p* < 0.05, vs. model group.

5-HT_1A_ mRNA in the hippocampus were measured using real-time quantitative PCR. The plasma 5-HT content was tested by HPLC-MS. Compared with the model group, 5-HT_1A_ mRNA levels were increased nearly three-fold in the Tiansi-treated group (*p* < 0.05) (Figure 3A). The plasma 5-HT content was lower in the model group than in the control group (*p* < 0.05), and it was significantly higher in the Tiansi-treated group than in the model group (*p* < 0.05) (Figure 3B). Thus, Tiansi Liquid promotes 5-HT_1A_ receptor expression in the hippocampus and significantly increases the concentration of 5-HT in the plasma of depressive rats.

### 2.4. The Effect of Tiansi Liquid on Microbiota Composition

Previous studies focused on fecal samples, and fecal microbiota has always been a marker of colonic microbiota. However, the distribution of bacteria in the small intestine and colon is quite different [27]. Experimental studies showed that psychological stress stagnates normal small intestinal transit time, drives the overgrowth of bacteria, and compromises the intestinal barrier [28]. Moreover, mucosa-associated microbiota differs from the microbiota present in the intestinal lumen [29], it may exert a more prominent effect on microbe–host interaction and affect the intestinal barrier function [30]. Therefore, besides the feces from the colon samples, we also chose the small intestine mucosa specimens to explore whether the change of small intestinal mucosa microbiota composition is related to the development of depression.

**Figure 4:**
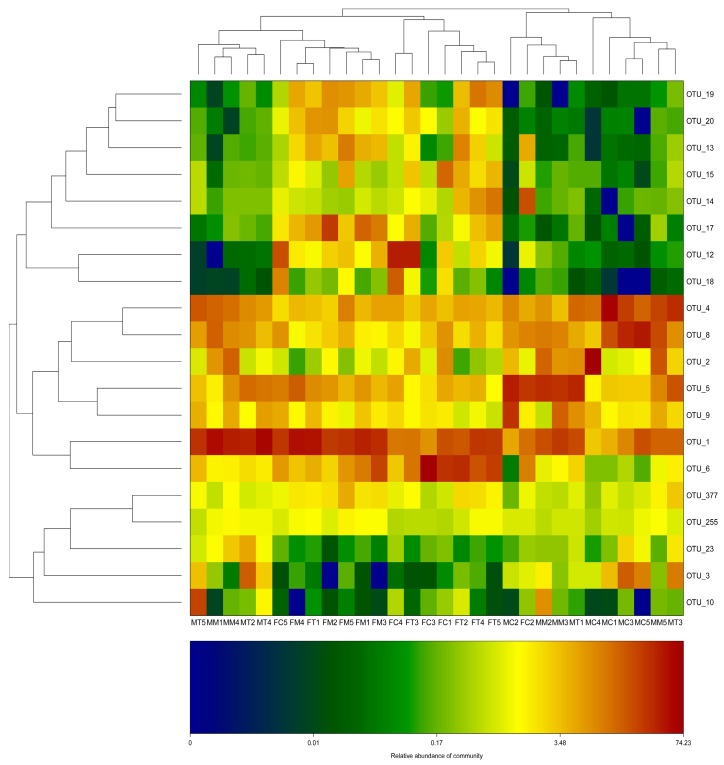
Distribution of OTUs in intestinal mucosa and fecal samples plotted as a heat map. The data were normalized to proportional abundance and are represented from low (blue) to high (red) for each OTU. OTUs indicate operational taxonomic units. FC, FM, and FT denote the control group, the model group, and the Tiansi-treated group of fecal samples, respectively; MC, MM and MT denote the control group, the model group and the Tiansi-treated group of intestinal mucosa samples, respectively.

The number of effective tags and OTUs observed after sequencing are shown in Table S1. The relative abundance of OTUs from fecal and intestinal mucosa samples were plotted as a heat map (Figure 4). The FC, FM and FT (fecal sample) groups were basically clustered, indicating that the overall microbiota composition is similar among the three groups and that Tiansi Liquid does not change the overall fecal microbiota structure. For the intestinal mucosa samples, some components in the MT and MM groups were clustered together, while some other MT and MC components were clustered in another subset, indicating that the overall structure of the intestinal mucosa microbiota was not identical among the three groups and that Tiansi Liquid may positively modify the intestinal mucosa microbiota structure in rats.

**Figure 5:**
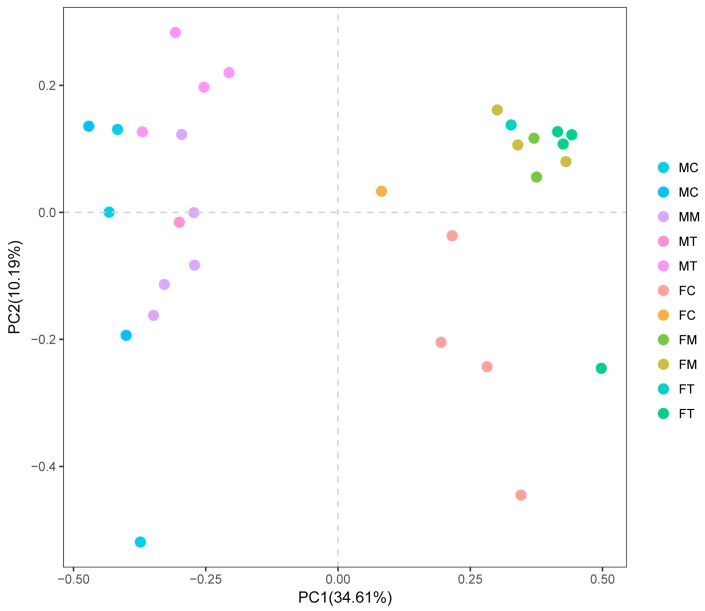
PCA plot of the Morisita–Horn dissimilarity matrix. PCA denotes principal component analysis.

The β-diversity of each group was analyzed by PCA and was calculated using QIIME, based on the relative abundance of OTUs in the fecal and intestinal mucosa samples from the control, model and Tiansi-treated groups (Figure 5). FM and FT were clustered together in the fecal samples, while FC was distant to these, indicating that the fecal microbiota of depressive rats was changed compared with the control rats and that Tiansi Liquid did not significantly change the fecal microbiota structure of depressive rats. For the intestinal mucosa samples, the MM, MC and MT groups were not clustered together, indicating that Tiansi Liquid changed the intestinal mucosa microbiota structure. The results were consistent with the heat map.

#### 2.4.1. Tiansi Liquid Alters Microbiota Diversity and Richness in Rats

**Figure 6:**
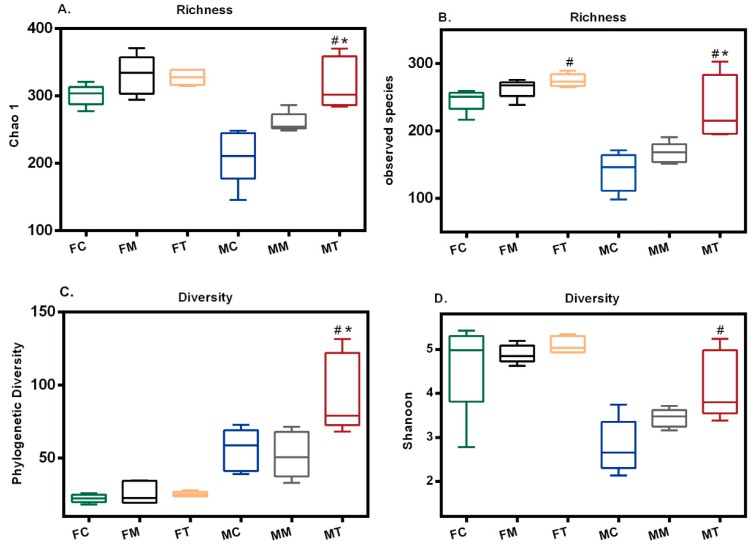
Tiansi Liquid treatment alters gut microbiota richness and diversity in fecal and intestinal samples. Chao 1 (**A**), observed_species (**B**), Phylogenetic Diversity (**C**), Shanoon (**D**). Box pots display the first (25%) and third (75%) quartiles, the median and the maximum and minimum observed values within each data set. There were five rats in each group. Statistical analyses were performed using one-way ANOVA and Tukey—Kramer post hoc comparisons. FC, FM and FT denote the control, model and Tiansi-treated groups of the fecal samples, respectively; and MC, MM and MT denote the control, model and Tiansi-treated groups of intestinal mucosa samples, respectively. ^#^ *p* < 0.05, vs. control group; * *p* < 0.05, vs. model group.

Species richness and diversity were determined from the number of bacterial species assigned by operational taxonomic units (OTUs) detected in the fecal and intestinal mucosa samples (Figure 6). Richness was estimated using the Observed species and Chao1 indices, and diversity was estimated by Phylogenetic diversity and Shannon.

In the fecal samples, species richness was significantly higher in the Tiansi group than in the control group, as indicated by the Observed species index (F (2, 12) = 5.447, *p* = 0.0207) (Figure 6B). There were no other significant differences among the three groups (Figure 6A,C,D). The trend of species richness in the model group was similar to that in the control and Tiansi-treated groups based on the rarefaction analysis estimates (Figure S2).

In the intestinal mucosa samples, species richness was significantly higher in the Tiansi-treated group than in the control and model groups, as indicated by Chao 1 (F (2, 12) = 13.04, *p* = 0.0010) and Observed species (F (2, 12) =10.95, *p* = 0.0020) indices (Figure 6A,B). Phylogenetic diversity was significantly higher in the Tiansi-treated group than in the control and model groups (F (2, 12) = 6.618, *p* = 0.0160) (Figure 6C), while Shannon was significantly higher in the Tiansi-treated group than in the control group (F (2, 12) = 6.986, *p* = 0.0097) (Figure 6D). The trend of species richness in the Tiansi group was higher than in the intestinal mucosa samples from the control and model groups, based on the rarefaction analysis estimates (Figure S2).

Although Tiansi Liquid did increase the microbiota richness and diversity in intestinal samples, collectively, these results suggest that the antidepressant-like effect of Tiansi Liquid may not be via its modification of microbiota richness and diversity. 

#### 2.4.2. Altered Microbiota Composition in the Depression Model

##### Microbiota Composition of Fecal Samples

**Figure 7:**
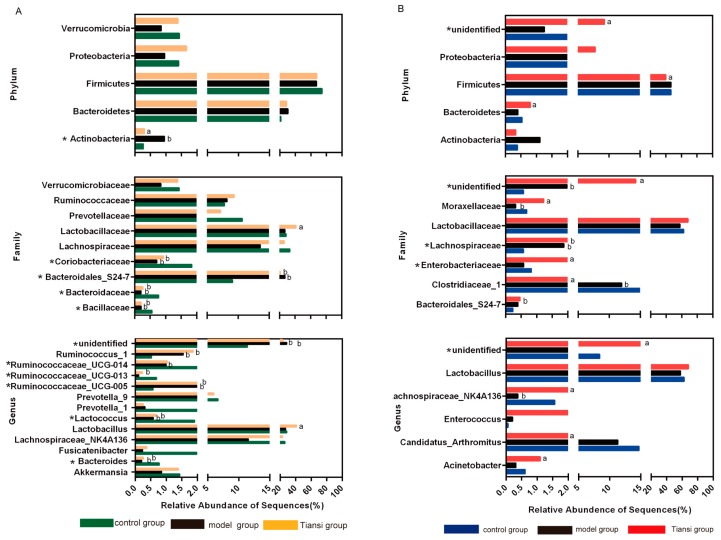
Taxonomic differences in fecal microbiota (**A**) and intestinal mucosa microbiota (**B**) among the control, model and Tiansi-treated groups. There were significant phylum, family and genus-level differences among these groups. A Kruskal–Wallis test identified the most differentially abundant taxa among the groups. * *p* < 0.05. Metastats was used for multiple comparisons of relative abundance differences of the samples and corrected with an FDR-adjust *p*-value ≤ 0.1 considered significant ^a^ *p* < 0.1, vs. model group; ^b^ *p* < 0.1, vs. control group.

We used Kruskal–Wallis analysis to investigate the associations of the fecal microbiota among the control, model and Tiansi-treated groups. The information on data distribution was in the supplementary file. Metastats was used for multiple comparisons of relative abundance differences of the samples and corrected with an FDR-adjust *p*-value ≤ 0.1 considered significant. Actinobacteria (*p* = 0.009) showed significant differences among the three groups at phyla level. The relative proportion of Actinobacteria (0.95% vs. 0.26%, *p* < 0.1) was markedly higher in the model group than in the control group (Figure 7A). We found four statistically significant differences (Coriobacteriaceae, *p* = 0.009; *Bacteroidales_S24-7_group*, *p* = 0.006; Bacteroidaceae, *p* = 0.007; Bacillaceae, *p* = 0.009) among the three groups at the family level. The relative proportions of Coriobacteriaceae (0.71% vs. 1.84%, *p* < 0.1), Bacteroidaceae (0.20% vs. 0.77%, *p* < 0.1) and Bacillaceae (0.20% vs. 0.56%, *p* < 0.1) were lower in the model group compared with the control group. We also found that the Lactobacillaceae family was more abundant in the Tiansi-treated group than in the model group (41.04% vs. 26.97%, *p* < 0.1) (Figure 7A). Bacterial communities were also compared at the genus level. The abundance of 75 genera in fecal samples differed among the control, model and Tiansi-treated groups, including 13 predominant (>0.5% of the total sequences in every group) and 62 sub-predominant genera. Five genera (*Bacteroides*, *p* = 0.007; *Lactococcus*, *p* = 0.012; *Ruminococcaceae_UCG-005*, *p* = 0.019; *Ruminococcaceae_UCG-013*, *p* = 0.015; *Ruminococcaceae_ UCG-014*, *p* = 0.03) were statistically significant differences among the three group. Among the four predominant genera, *Bacteroides* (0.20% vs. 0.77%, *p* < 0.1), *Lactococcus* (0.57% vs. 1.91%, *p* < 0.1), *Ruminococcaceae_UCG-013* (0.10% vs. 0.68%, *p* < 0.1) and *Ruminococcaceae_UCG-014* (1.00% vs. 2.40%) were less abundant in the model group than in the control group. *Lactobacillus* (41.04% vs. 26.97%, *p* < 0.1) was more abundant in the Tiansi-treated group than in the model group (Figure 7A).

##### Microbiota Composition of Intestinal Mucosa Samples

At the phylum level, the relative abundance of Firmicutes (72.60% vs. 91.16%, *p* < 0.1) was lower in Tiansi group than in the model group, while Bacteroidetes (1.37% vs. 0.56%, *p* < 0.1) was higher. Enterobacteriaceae (*p* = 0.045) and Lachnospiraceae (*p* = 0.004) showed significant differences among the three groups at family level (Figure 7B). Compared with the control group, the abundance levels of the following more prevalent families were decreased in the model group: Clostridiaceae_1 (12.02% vs. 15.33%, *p* < 0.1), Moraxellaceae (0.32% vs. 0.67%, *p* < 0.1); while Lachnospiraceae (1.87% vs. 0.57%, *p* < 0.1) was increased in the model group. We also found that the following families were more abundant in the Tiansi-treated group than in the model group: Enterobacteriaceae (2.41% vs. 0.58%, *p* < 0.1); Moraxellaceae (1.23% vs. 0.32%, *p* < 0.1) (Figure 7B). *Lachnospiraceae_NK4A136_group* (*p* = 0.003) was significant difference among the three groups were observed at the genus level as. The relative abundance of *Lachnospiraceae_NK4A136_group* (0.38% vs. 1.57%, *p* < 0.1) was decreased in the model group compared with the control group. Compared with the model group, Tiansi Liquid treatment greatly increased the relative abundance of *Lachnospiraceae_NK4A136_group* (2.67% vs. 0.20%, *p* < 0.1) and *Acinetobacter* (1.10% vs. 0.31%, *p* < 0.1), and decreased the relative abundance of *Candidatus_Arthromitus* (2.08% vs. 11.33%, *p* < 0.1).

### 2.5. The Associations among Microbiota and Tryptophan Metabolism

**Figure 8:**
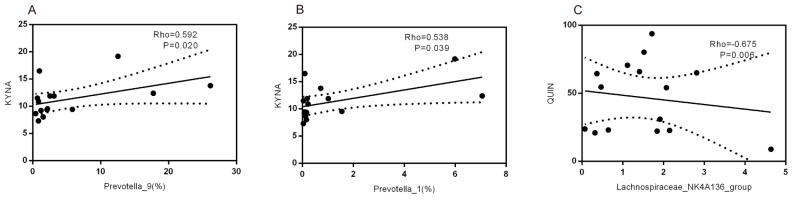
Correlation between KYNA content and the relative abundance of the genera, Precotella_9 (**A**) and Prevotella_1 (**B**). Correlation between QUIN content and abundance of the genus, *Lachnospiraceae_NK4A136_group* (**C**). Spearman rank correlation (R) and probability (*p*) were used to evaluate statistical significance.

In the Spearman rank correlation analysis, the predominant genera (>0.5% of the total sequences in every group), and the results of metabonomics of corresponding numbered rats were selected. We found that several altered genera were correlated with altered tryptophan metabolism. The relative abundance of Prevotella and KYNA content displayed a significant positive correlation with Tiansi Liquid treatment (Figure 8A,B). Meanwhile, there was a negative correlation between the relative abundance of the *Lachnospiraceae_NK4A136_group* and QUIN content (Figure 8C).

## 3. Discussion

About 92% of depressive patients experience stressful events before the onset of the disease [31]. The use of a reliable stress-induced depression model was critical for our study. We produced such a model by injecting hydrocortisone for 21 days in rats, which results in symptoms similar to that elicited by restraint-induced stress in rodents [32]. In our study, treatment with Tiansi Liquid greatly increased the sucrose preference as well as exploratory behavior and activity in this rat model of depression. This suggests that Tiansi Liquid has an antidepressant-like effect.

A previous study suggested that depression is associated with a decreased richness and diversity of gut microbiota [33]. However, another study reported no obvious differences in microbiota diversity between depressed and non-depressed individuals [8]. Our current findings show that there was no significant change in microbiota structure among the control, model and Tiansi-treated groups in the fecal samples. In contrast, in the intestinal mucosa samples, the microbiota structure changed among the three groups. Compared with normal rats, the richness and diversity increased significantly in depressive rats, and Tiansi Liquid treatment further enhanced the intestinal mucosa microbiota diversity. Collectively, the antidepressant-like effect of Tiansi Liquid may not be via its modification of microbiota richness and diversity.

In this work, the microbiomes of fecal and intestinal mucosa samples in the multiple comparisons were characterized by remarkable taxonomical differences in the three phyla—Firmicutes, Bacteroidetes and Actinobacteria. Firmicutes and Bacteroidetes are the predominant phyla in the gut of animals and humans [34], while Actinobacteria are minor phyla [35]. Germ-free mice can be induced depression by transplantation of Actinobacteria bacteria [36].

Fusicatenibacter and Candidatus_Arthromitus belong to the Bacteroidaceae family, while Prevotella_9 and Prevotella_1 belong to the Prevotellaceae family. These two families contributed primarily to the overall increase in Bacteroidetes. It has been suggested that Candidatus Arthromitus is positively correlated with 3-methyldioxyindole and can be modulated by this tryptophan metabolite [37]. We found that the abundance of Candidatus Arthromitus in the rats’ model of depression was decreased and that Tiansi Liquid treatment did not affect this species (Figure 7). Higher proportions of Prevotella in fecal microbial communities may serve as disease signatures for patients with major depressive disorders [38,39], while other studies founded that Prevotella was the lower in the major depressive patients than in the healthy controls [40]. In our study, the relative abundance of Prevotella has a decreasing trend in the model group without significant difference, compared with control group. This situation may be caused by the small size (five rats in each group) in microbiota analysis, it also the limitation in our study. Meanwhile, Prevotella can affect the serum metabolomics in different disease models, such as diabetes and hyperlipidemia [41,42]. From the Spearman correlation analysis, we demonstrated that Prevotella_1 and Prevotella_9 were significantly correlated with KYNA levels (Figure 8), It suggests that Prevotella may affect serum metabolism in depression model.

It was previously reported that the various species of Lactobacillaceae (*Lactobacillus rhamnosus JB-1*, *Lactobacillus helveticus R0052* and *Lactobacillus plantarum strain PS128*) are helpful for alleviating the symptoms of depression [43,44,45]. Furthermore, *Lactococcus* can prevent the colonization of certain commensal microorganisms [46]. Here, Tiansi Liquid treatment greatly increased the proportion of these bacteria, consistent with their proposed beneficial role.

*Ruminococcaceae* is a family in the class, Clostridia, which consumes tryptophan and converts it to tryptamine [11,47]. Some of the *Ruminococcaceae* exert antidepressant effects [41]. *Enterococcus* is an opportunistic pathogen that is widespread in the intestine and is used in certain probiotic preparations [48,49]. In the present study, Tiansi Liquid treatment significantly altered the ratio of *Ruminococcus* (fecal samples) and *Enterococcus* (intestinal mucosa samples) in the rat model of depression. Tiansi Liquid treatment also greatly increased the relative abundance of *Lachnospiraceae_NK4A136_group* in the feces and intestine. The expression of Lachnospiraceae and Ruminococcaceae in mice correlated with depressive behavioral changes induced by stress [50]. In summary, we found Tiansi Liquid could regulated the relative abundance of some microbiota in intestinal mucosa or fecal samples, which were related with depression.

Perturbation of the TRP–KYN pathway is found in both depressed patients and in animal models of depression [51,52]. Some metabolites of tryptophan such as QUIN are neurotoxic while KYNA is neuroprotective [18]. Stress-inducible TDO, which is activated by glucocorticoids, is predominantly expressed in the liver and is the main source of KYN under non-inflammatory conditions [53]. In addition, chronic stress increases the expression of IDO by enhancing pro-inflammatory cytokines and thereby, in turn, enhancing KYN production [54]. Glucocorticoid administration may have induced activation of TDO, leading to the degradation of tryptophan [55,56] and decreased central presynaptic 5-HT levels, resulting in a depressive state [57,58]. In our study, Tiansi Liquid treatment significantly inhibited the expression of TDO mRNA and IDO mRNA, reduced the KYN/TRP ratio and the amounts of QUIN and KYN, while significantly increasing the levels of KYNA and 5-HT. These results suggest that Tiansi Liquid treatment ameliorates the symptoms of depression by modulating the TRP–KYN pathway.

There are reports that hydrocortisone treatment may lead to the altered release of other factors that have been linked to depression, such as amyloid beta (Aβ) [59], which has been shown to modulate the HPA axis to induce a depressive-like phenotype and alter kynurenine levels [60,61]. Interestingly, Tiansi Liquid was reported to significantly improve memory impairment and neuronal damage in a rat model of Alzheimer’s disease produced by injecting Aβ_25–35_ [62].

Tryptophan is a major substrate in microbial metabolic pathways [63], and microbes may exert their beneficial effects on mood by regulating the TRP–KYN metabolic pathway [20] and thereby elevating 5-HT levels. Previous studies show that the genus, *Prevotella*, is related to levels of tryptophan [64,65]. Furthermore, the *Ruminococcus* was positively correlated with tryptophan metabolism. Ruminoccus gnavus produces tryptamine by consuming tryptophan [66]. These observations are in line with our current finding that Tiansi Liquid greatly increases the relative abundance of microbiota (Lachnospiraceae and Ruminococcaceae) related to the TRP–KYN metabolic pathway, as well as other microbiota (*Lactococcus* and *Lactobacillus*) related to depression. From the Spearman correlation analysis, we demonstrated that Prevotella_1 and Prevotella_9 were significantly correlated with KYNA levels (Figure 8A,B). Meanwhile, there was a negative correlation between the relative abundance of *Lachnospiraceae_NK4A136_group* and the content of QUIN (Figure 8C). Further studies are needed to testify whether Lachnospiraceae has influence on the content of QUIN in the depression model.

## 4. Materials and Methods

### 4.1. Animals and Treatments

Adult male Sprague-Dawley rats weighing 250–280 g were acquired from the center of laboratory animal science of the Academy of Military Medical Sciences of the Chinese People’s Liberation Army. (License number: SCXK (JING) 2012-0004). All rats were housed individually at a constant temperature (21 ± 1 °C) in a specific pathogen-free environment and maintained on a 12 h light/dark cycle. They were adapted for one week, with free access to food and water.

All the experimental procedures involving animals were approved by the Sub-Committee of Experimental Animal Ethics, Academic Committee of Beijing University of Chinese Medicine (1100000013479; project identification code: BZYYYDX-LL-20160301).

### 4.2. Generation of the Rat Model of Depression

Rats were randomly divided into the control, model and Tiansi groups, with ten rats in each group. A stress-induced depression model was established by intraperitoneally injecting hydrocortisone (H0533-25G, TCI, Shanghai, China) dissolved in 0.2% Tween 80 and 0.2% DMSO/normal saline, at 40 mg/kg once a day for 21 consecutive days prior to the behavioral testing [67,68]. Male rats were employed to avoid variable steroids levels observed of the female rats during the regular diestrus [69]. The control and model groups were given normal saline injection, while the Tiansi group was administered Tiansi Liquid. The dose of Tiansi Liquid was 0.45 g/kg once a day [25].

### 4.3. Preparation of the Extract

Tiansi Liquid was made using *Morinda officinalis* How polysaccharides and *Cuscuta chinensis* polysaccharides at a ratio of 1:1. The specific method for obtaining the polysaccharides from *Morinda officinalis* How and *Cuscuta chinensis* has been described previously [70,71], and is summarized in the Supplementary Materials. The model rats were administrated Tiansi Liquid by means of intragastric administration between 9–11 a.m. every morning.

### 4.4. Sample Preparation

Previous studies focused on fecal samples. We collected both the feces from the colon samples and the small intestinal mucosa samples for this study. Feces and small intestinal mucosa were collected for the sequence analysis of 16S rRNA. The intestinal contents were isolated and suspended in nine volumes of cold phosphate-buffered-saline (PBS). Liver and hippocampal tissues were used for PCR analysis. All samples were collected on day 21, then quickly snap-frozen in liquid nitrogen and kept at −80 °C until analysis.

### 4.5. Behavior Testing

#### 4.5.1. Sucrose Preference Test (SPT)

The SPT was performed on days 7, 14 and 21. It was performed in two phases and at intervals of 12 h. For the training phase, ten rats in each group were habituated for 12 h to the presence of two drinking bottles in their cage. After the training phase, the rats were fasted for 12 h and presented with two drinking bottles for 3 h in their cages, one containing 1% sucrose and the other containing water. Water and sucrose solution intake were recorded by measuring the bottles for 3 h. Sucrose preference was calculated as a percentage of the volume of sucrose intake to the total volume of fluid intake. Sucrose preference = V (sucrose solution)/[V (sucrose solution) + V (water)] × 100% [72].

#### 4.5.2. Open Field Test (OFT)

The OFT measures the general locomotive behavior of the animal and is issued to ensure that any change observed in the mobility of the animals in the forced swimming test (FST) is not due to general alterations in locomotor activity but rather has a psychogenic origin. The OFT scores were calculated as the sum of the horizontal and vertical motion scores to assess the overall exploratory behavior and activity. The OPT was performed on days 7, 14 and 21. The open field was a square empty environment, with the following dimensions: 100 cm × 100 cm × 40 cm (length × width × height). A central area was drawn in the open field. It was centered in the center of the empty field, with a square length of 16 cm. The animals were placed in the middle of the central area, then observed for three minutes. Their movement was recorded on video. The total distance moved (cm) was scored using EthoVision XT software (Noldus Information Technology, Wageningen, The Netherlands). The open field apparatus was cleaned after each session using 70% ethyl alcohol, and permitted to dry between tests [73].

### 4.6. Plasma TRP–KYN Pathway Analysis

Plasma samples of ten rats in each group were analyzed for TRP, KYN, KYNA, QUIN and 5-HT. Abdominal aortic blood was collected and kept in a refrigerator at 4 °C. The blood samples were centrifuged at 2500 rpm for 15 min following coagulation, and then a 100 µL aliquot of supernatant was collected and stored in a −20 °C freezer. The detection of content (TRP, KY, KYNA, QUIN and 5-HT) was performed using high-performance liquid chromatography-mass spectrometry (HPLC-MS/MS).

#### 4.6.1. HPLC-MS/MS Conditions

HPLC separation was performed on Slab HP-C18 columns (150 × 4.6 mm, 5 µm). Injection volumes were 20 µL for all samples. The mobile phase contained solvents A and B, where A was water and B was acetonitrile. The linear gradient program for abdominal aortic blood was: 95% B from 0 to 1 min; 95–40% B from 1 to 8 min; 40–0% B from 8 to 8.1 min; 0–95% B from 10 to 10.1 min; and 95% B from 10.1 to 15 min. The flow rate was 0.8 mL/min for HPLC-MS/MS, with the column temperature maintained at 30 °C.

Mass spectra were acquired on a SCIEX Ultimate 3000-API 3200 QTRAP TOF mass spectrometer (ABSCIEX, Boston, Massachusetts, USA) combined with an ESI source in positive ion scan mode. The parameters were set as follows: Capillary voltage, 5.5 kV; desolvation temperature, 400 °C; atomization gas velocity, 55 psi; auxiliary gas, 60 psi; injection voltage, 10; and collision ejaculated voltage, 2 [74].

#### 4.6.2. PCR Analysis

TDO mRNA in the liver, and IDO mRNA and 5-HT_1A_ mRNA in the hippocampus were measured using real-time quantitative PCR. Livers were dissected, and the total RNA was isolated using Trizol reagent (No.155W026, Invitrogen, Carlsbad, CA, USA), and the cDNA was synthesized using a HiFi-MMLV cDNA Kit (No. CW0744, Cwbio, Beijing, China). PCR reactions were performed using a CFX96 with Power SYBR^®^ Green JumpStart Taq ReadyMix (No. CW0W7A, Cwbio). The PCR reaction conditions were as follows: 42 °C for 6 min, followed by 70 °C for 8 min; then 40 cycles of 95 °C for 15 s, 55 °C for 45 s, and fluorescence collection at 55 °C. A final extension was performed at 40 °C for 1 min. Relative quantification of each sample was calculated using 2^−ΔΔct^ and expressed as a percentage of control. The primer sequences were as follows: GAPDH-F: 5′ ATTGTCAGCAATGCATCCTG 3′; GAPDH-R: 5′ ATGGACTGTGGTCATGAGCC 3′; TDO-F: 5′ GGCTATTATTATCTGCGCTCAACTG 3′; TDO-R: 5′ AACCAGGTACGATGAGAGGTTAAA 3′; IDO-F: 5′ GACCCGAAAGCACTGGAGA 3′; IDO-F: 5′ TGCCCTTCCAACCAGACAA 3′; 5-HT_1A_-F: 5′ CTGCCCATGGCTGCTCTGTA 3′; and 5-HT_1A_-R: 5′ CATCCAGGGCGATAAACAGGTC 3′.

### 4.7. Sequence Analysis of 16S rRNA 

Five rats of each group were randomly employed into the sequence analysis of 16S rRNA. The gut bacterial composition in the fecal and intestinal mucosa of rats was analyzed by 16S rRNA gene analysis. The 16S rRNA genes were amplified using a specific primer, 338F-806R, to target the V3–V4 regions of 16S rRNA. PCR products were mixed in equal ratios and purified using the AxyPrep Gel Extraction Kit (Axygen, San Francisco, CA, USA). Sequencing libraries were generated using the NEBNext Ultra DNA Library Prep Kit for Illumina (New England Biolabs, Beijing, China) following the manufacturer’s recommendations, and index codes were added. The library quality was assessed using a Qubit 2.0 Fluorometer (Thermo Scientific, Shanghai, China) and an Agilent Bioanalyzer 2100 system (Agilent Technologies, Beijing, China). The library was sequenced on the MiSeq (Illumina) platform, and 300-bp paired-end reads were generated.

Sequences were analyzed using the QIIME software package (Quantitative Insights into Microbial Ecology, V1.8.0). First, the reads were filtered by the QIIME quality filters. Then, we picked a representative sequence for each OTU and used the RDP (Ribosomal Database Project) classifier to annotate taxonomic information for each representative sequence via the SILVA 119 ribosomal RNA (rRNA) database. Sequences with similarities over 97% were assigned to the same OTU.

### 4.8. Statistical Analysis

Statistical analyses were performed using one-way ANOVA, the Kruskal–Wallis test, metastats, spearman correlation analysis, as appropriate. *p* < 0.05 was considered statistically significant. Statistical analysis was performed using SPSS 20.0 software (IBM, Armonk, NY, USA). GraphPad Prism 6 (GraphPad Software Inc., San Diego, CA, USA) was used for generating graphs.

## 5. Conclusions

We investigated the effects of Tiansi Liquid on behavioral changes, microbial richness and diversity, the microbiota composition of intestinal and fecal samples, and the TRP–KYN pathway metabolites in a rat model of hydrocortisone-induced depression. We found that Tiansi Liquid greatly ameliorated the behavior of depressive rats. This behavioral improvement was associated with changes in metabolites in the TRP–KYN pathway and an increase in the expression of 5-HT mRNA in the hippocampus and 5-HT content in plasma. Furthermore, Tiansi Liquid increased the relative abundance of microbiota (*Lachnospiraceae* and *Ruminococcaceae)* related to the TRP–KYN metabolic pathway and of microbiota (*Lactococcus* and *Lactobacillus*) related to depression. Spearman correlation analysis revealed that some altered gut microbiota genera were strongly correlated with altered TRP–KYN metabolites. Despite the preliminary data, our novel findings suggest that Tiansi Liquid may alleviate the symptoms of depression by modulating the gut microbiota composition and TRP–KYN metabolic pathway.

## Supplementary Materials

The following are available online at http://www.mdpi.com/1420-3049/23/11/2832/s1, Table S1: Tiansi Liquid changed the bacterial communities of fecal and intestinal samples. Figure S1: A Venn diagram illustrating an overlap of OTUs in the fecal and intestinal microbiota. Figure S2: Rarefaction curves were used to estimate the richness of fecal and intestinal mucosa microbiota. Supplementary file 2: Further information about Tiansi Liquid. Supplement file 3: The information on data distribution of microbiota of Kruskal–Wallis analysis.
