# Supplementary material for: Tiansi Liquid Modulates Gut Microbiota Composition and Tryptophan–Kynurenine Metabolism in Rats with Hydrocortisone-Induced Depression
Source: Molecules. 2018 Oct 31;23(11):2832. doi: 10.3390/molecules23112832 (PMC6278342; doi:10.3390/molecules23112832)
Supplement: Supplementary file 1 [file molecules-23-02832-s001.zip › supplementary file/supplementary file 2.docx]

Tiansi Liquid was made using morida offcinalis how polysaccharide and cuscuta chinensis polysaccharide according to the ratio of 1:1, the specific method for the polysaccharides from Morinda officinalis how and Cuscuta chinensis relying on the following references, and the purity of cuscuta chinensis polysaccharide is precisely described by 13 C-NMR and HPLC (Xu et al.,2011; Liu, 2011).

1. Xu L.Y., Li Z.M., Yang L., Lv Y.L., Wang D., Li X.R.(2011). Quantitative determination of polysaccharides in Tusizi( Semen Cuscutae). J. Beijing Univ. Tradit. Chin. Med. 34, 548-551.

2. Liu J.J.(2011). Effect of Polysaccharides from Morinda Offcin alis on the Oxidative Stress and Congitive-behaviors in An Experimental Depression Model of Rats. China Modern Doctor. 49, 1-2,5.

Preparation of Polysaccharides from Cuscuta chinensis: Weighing a certain amount of Cuscuta chinensis precisely, then adding 10 times the volume of 80% ethanol, reflux extraction 2 times, 1 hour each time, degreasing, filtering. After volatilization of ethanol, the residues were extracted by boiling water 2 times, 2 hours each time, then centrifuging at 5000 R /min, 10 minutes. Combining with supernatant, concentrated, adding ethanol to make the alcohol content of 80%, at 4 ℃ refrigerator. Overnight, filtered and dried.

According to the results of literature [2], The main components of cuscuta chinensis polysaccharide are as follows: mannose, galactosidonic acid, glucose, galactose, xylose, arabinose. The content of active ingredients was 8.1%.

Preparation of morida offcinalis how polysaccharide: A suitable amount of Morinda officinalis rhizome was selected, and was dried at 60 ℃ to constant weight. Weighing a certain amount of morida offcinalis, grinding into powder, and add distilled water in the flask (W: W=1:25). Then heated to 75℃ for boiling 4h. After the extract was cooled, 95% ethanol (W: W=1: 4) was used to extract Morinda officinalis polysaccharides. The polysaccharides of Morinda officinalis were precipitated at 2℃ for 12 hours and centrifuged. The polysaccharides of Morinda officinalis were filtered by membrane, deproteinized and freeze-dried [1].
