# Supplementary material for: Tiansi Liquid Modulates Gut Microbiota Composition and Tryptophan–Kynurenine Metabolism in Rats with Hydrocortisone-Induced Depression
Source: Molecules. 2018 Oct 31;23(11):2832. doi: 10.3390/molecules23112832 (PMC6278342; doi:10.3390/molecules23112832)
Supplement: Supplementary file 1 [file molecules-23-02832-s001.zip › supplementary file/supplementary information-edited.docx]

***Supplementary Material***

**Tiansi Liquid Modulates Gut Microbiota Composition and Tryptophan–Kynurenine Metabolism in Rats with Hydrocortisone-Induced Depression**

Dan Cheng ^1,†^, Hongsheng Chang ^2,†^, Suya Ma ^1^, Jian Guo ^1^, Gaimei She ^2^, Feilong Zhang ^1^, Lingling Li ^1^, Xinjie Li ^1^ and Yi Lu ^1,^*

^1^ School of Chinese Medicine, Beijing University of Chinese Medicine, Beijing 100029, China; [chengdan@bucm.edu.cn (D.C.)](mailto:chengdan@bucm.edu.cn%20(D.C.)) ; [masuya0217@163.com](mailto:masuya0217@163.com) (S.M.); [guojian323@sina.com](mailto:guojian323@sina.com) (J.G.); delko@bucm.edu.cn (F.Z.); [20170931145@bucm.edu.cn](mailto:20170931145@bucm.edu.cn) (L.L); [lixinjie@bucm.edu.cn](mailto:lixinjie@bucm.edu.cn) (X.L.); [luyi@bucm.edu.cn](mailto:luyi@bucm.edu.cn) (Y.L.)

^2^ School of Chinese Materia Medica, Beijing University of Chinese Medicine, Beijing 102488, China; [chs1971@sina.com](mailto:chs1971@sina.com) (H.C.); [shegaimei@126.com](mailto:shegaimei@126.com) (G.S.);

***** Correspondence: [luyi@bucm.edu.cn](mailto:luyi@bucm.edu.cn); Tel.: +86-64-286-576

† These authors contributed equally to this work.

**Table S1**. Tiansi Liquid changed the bacterial communities of fecal and intestinal samples.

| Sample | Final_tags | OTUs | Number of different taxonomic categories | | | | |
| --- | --- | --- | --- | --- | --- | --- | --- |
|  |  |  | Phylum | Class | Order | Family | Genus |
| FC | 18059 | 398 | 10 | 21 | 31 | 53 | 119 |
| FM | 11506 | 404 | 10 | 21 | 31 | 50 | 108 |
| FT | 11109 | 404 | 10 | 21 | 30 | 48 | 108 |
| MC | 21267 | 450 | 10 | 21 | 30 | 53 | 128 |
| MM | 17539 | 457 | 11 | 22 | 32 | 56 | 122 |
| MT | 18198 | 610 | 11 | 21 | 31 | 61 | 124 |

**Note**:① Samples of FC、FM、FT indicate control group, model group and Tiansi group respectively of fecal samples; samples of MC、MM、MT indicate control group、model group and Tiansi group respectively of intestinal samples.


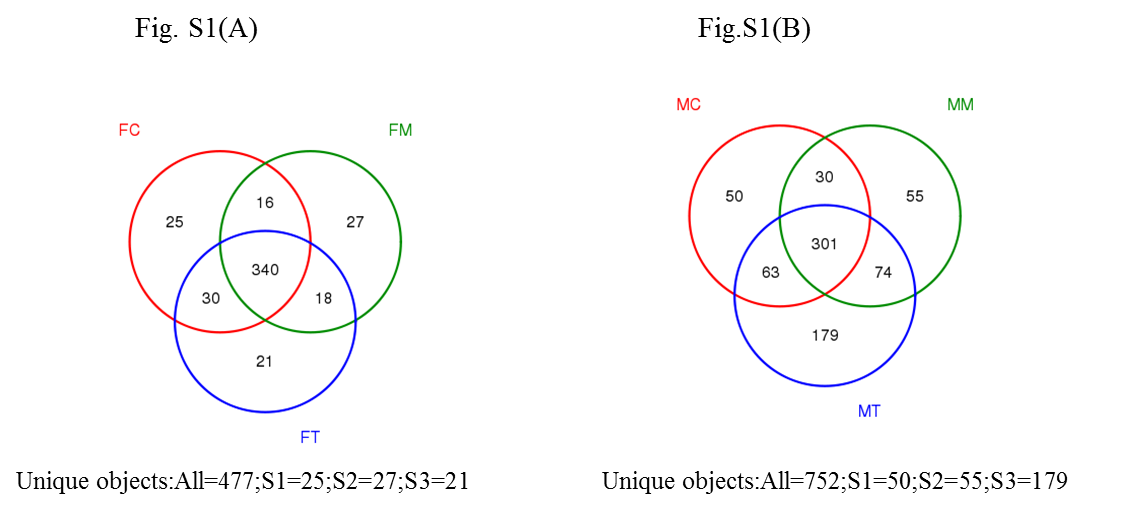


**Figure S1**. A Venn diagram illustrating overlap of OTUs in fecal (A) and intestinal microbiota(B). FC, FM, and FT indicate the control group, the model group, and the Tiansi-treated group of fecal samples; MC, MM, and MT indicate the control group, the model group, and the Tiansi-treated group of intestinal samples.


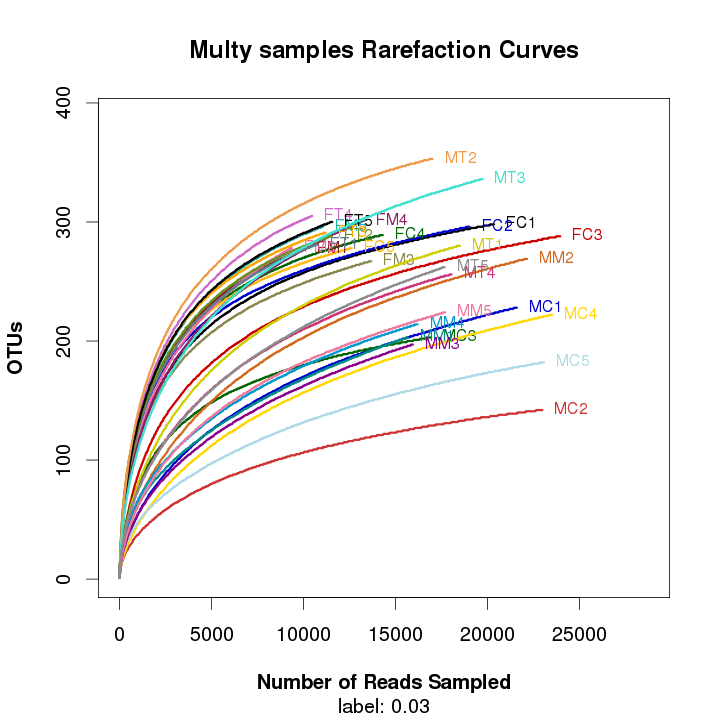


**Figure S2.** Rarefaction curves were used to estimate the richness of fecal and intestinal mucosa microbiota. In this case the number of taxa at a 97% similarity level The vertical axis shows the number of OTUs that would be expected to be found after sampling the number of tags or sequences shown on the horizontal axis. FC, FM, and FT indicate the control group, the model group, and the Tiansi-treated group of fecal samples; MC, MM, and MT indicate the control group, the model group, and the Tiansi-treated group of intestinal samples.
